# Supplementary material for: Sequence learning, prediction, and replay in networks of spiking neurons
Source: PLoS Comput Biol. 2022 Jun 21;18(6):e1010233. doi: 10.1371/journal.pcbi.1010233 (PMC9273101; doi:10.1371/journal.pcbi.1010233)
Supplement: Attachment — Submitted filename: PCOMPBIOLD210201_review.pdf [file pcbi.1010233.s011.pdf]

Review of “An exploratory analysis of hippocampus-amygdala interactions”

In the Manuscript ‘An exploratory analysis of hippocampus-amygdala interactions’ the Authors propose a reformulation of the Hierarchical Temporal Memory (HTM) algorithm in terms of leaky integrate-and-fire recurrent neural networks. The proposed work aims to add biophysical realism to the original algorithm by reformulating its discrete-time dynamics with a continuous-time network and by introducing biologically plausible plasticity mechanisms such as structural Hebbian and homeostatic plasticity. The proposed network is able to learn and predict high-order sequences. Moreover, when increasing its excitability, the network replays the learned sequences at a faster speed. Finally, the Authors investigate how the timing at which the external inputs occur in the sequence impacts the prediction performance.

The Manuscript is well written and easy to follow. I appreciated the clear tables describing the network structure and the detailed report of the network parameter used. The Manuscript addresses two important topics such as that of sequence learning and replay, and that of prediction error in learned sequences of stimuli. I have however two major concerns (in particular the first listed here) that limit the impact of the proposed Manuscript:

Major points:

1) The main goal of the Manuscript is to translate a highly abstract and artificial algorithm, such as that of the HTM, into a biologically plausible neural network. As the Authors write at the end of the abstract “By strengthening the link to biology, our implementation facilitates the evaluation of the TM hypothesis based on experimentally accessible quantities”. Despite the clear effort in translating the TM algorithm into spiking neuronal networks, I found some aspects of the proposed network still far from biologically plausible:
    a. In the proposed model, input stimuli are passed to the network by 1 single spike. While theories on temporal coding (as opposed to rate coding) acknowledge the relevance of single spikes timing for neural computation, it is undisputed that stimulus responses in most brain regions are also rate modulated. The absence of rate modulations in either network inputs or network responses is a very strong abstraction and limits the scope of the paper, especially considering the intent of the paper to link the HTM model to biology.
    b. In the proposed model there is no background activity and the spiking statistics and variability produced by the network (visible in the rasterplots) look far from what one observes experimentally.

    Both these two features are highly relevant and, given the presence of plasticity and the feedback inhibitory loop, might significantly alter the model dynamics. To facilitate biological interpretation it would therefore be important to show that the network can learn and reproduce high-order stimulus sequences also after the introduction of these two features.

2) There is a vast literature on: a) neural networks able to produce sequences, b) networks able to produce replays (backward and forward). Surprisingly the section on ‘Relationship to other models’ doesn’t address this literature, as one would expect, except for the sentence “This is in essence similar to several other spiking neuronal network models for sequence learning [9–12].”. It would be

useful to add a paragraph where other models are discussed and compared with the proposed model.

Minor points:

3) The model has one inhibitory neuron for each excitatory subnetwork. Fig 2A,C could lead to interpret the inhibitory network as recurrently coupled with the full network of excitatory neurons. I would suggest to slightly modify the scheme and add as many inhibitory neurons as the excitatory subnetworks, each of them connected only to their respective excitatory subgroup.

4) There is a probable typo at line 236: "… synaptic depression, and is triggered by each **presynaptic** spike …", Most probably the authors mean **postsynaptic**.

5) Line 137: "connections can be either "mature" or "immature" ("effective" or "potential"; see below)". For clarity, I would stick with one of the two definitions throughout the manuscript (either mature/immature or effective/potential). Also, immature/potential connections, although updated, do not affect units activity when running the network. Even though this is deducible from lines 197-198, it is not evident from the rest of the paper. I would suggest adding this information explicitly, at least at line 137, when the two types of connections are introduced.

6) When referring to "relative number of active neurons", e.g. legend of Fig. 8C, to my understanding the authors refer to the relative number of active neurons of the targeted population. If this is the case, it would be useful to add this information in the label of the plot axis or at least in the figure legend.
