# Supplementary material for: Sequence learning, prediction, and replay in networks of spiking neurons
Source: PLoS Comput Biol. 2022 Jun 21;18(6):e1010233. doi: 10.1371/journal.pcbi.1010233 (PMC9273101; doi:10.1371/journal.pcbi.1010233)
Supplement: Attachment — Submitted filename: reply_letter.pdf [file pcbi.1010233.s012.pdf]

Younes Bouhadjar
Institute of Neuroscience and
Medicine (INM-6) &
Peter Grünberg Institut (PGI-7)
Jülich Research Centre
D-52425 Jülich
Germany

phone: +491778728402
email: y.bouhadjar@fz-juelich.de

Stefan Kiebel and Samuel Gershman
Editorial Board Member, PLoS Computational Biology

Jülich
February 17, 2022

Resubmission of manuscript PCOMPBIOL-D-21-02013

Dear Stefan Kiebel and Samuel Gershman,

please find enclosed our revised manuscript *"Sequence learning, prediction, and replay in networks of spiking neurons"*, which we resubmit for publication in PLoS Computational Biology. All concerns of the reviewers have been addressed, and the manuscript was changed accordingly. A point-by-point reply for each reviewer is provided below on separate pages.

As requested, we resubmit two versions of the revised manuscript, one with highlighted changes and references to the respective reviewer comments, and one without change tracking information.

Best regards,

Younes Bouhadjar (on behalf of all authors)

encl
- reply to reviewer #1
- reply to reviewer #2
- revised manuscript with highlighted changes
- revised manuscript without change highlighting

Reply to comments of reviewer #1

[R1:1] *Major points:*
The main goal of the Manuscript is to translate a highly abstract and artificial algorithm, such as that of the HTM, into a biologically plausible neural network. As the Authors write at the end of the abstract "By strengthening the link to biology, our implementation facilitates the evaluation of the TM hypothesis based on experimentally accessible quantities". Despite the clear effort in translating the TM algorithm into spiking neuronal networks, I found some aspects of the proposed network still far from biologically plausible:

We thank the reviewer for the valuable comments and the constructive critique. In the revised version of the manuscript, we have addressed all the reviewer's concerns, and marked all revised parts by color and a reference "[R1:X]" to the corresponding comment X. A point-by-point reply is provided below.

[R1:2] *a. In the proposed model, input stimuli are passed to the network by a single spike. While theories on temporal coding (as opposed to rate coding) acknowledge the relevance of single spikes timing for neural computation, it is undisputed that stimulus responses in most brain regions are also rate modulated. The absence of rate modulations in either network inputs or network responses is a very strong abstraction and limits the scope of the paper, especially considering the intent of the paper to link the HTM model to biology.*

We agree that the role of synchrony requires further explanation. In the revised manuscript, we now address this point in the 3rd paragraph in "Discussion: Limitations and outlook", explaining that single input spikes mimic inputs from synchronously active populations of external neurons, and that this input synchrony is not strictly necessary provided the responses in the TM populations remain (nearly) synchronous. We further make the point that the overall network dynamics could possibly be preserved even if the TM populations fired in a non-synchronous and rate modulated manner, but that this would lead to a loss of sparsity, which is one of the key advantages of the architecture proposed by Hawkins & Ahmad (2016).

[R1:3] *b. In the proposed model there is no background activity and the spiking statistics and variability produced by the network (visible in the raster plots) look far from what one observes experimentally.*

We agree and thank the reviewer for giving us the opportunity to elaborate on this. In a new paragraph added to "Discussion: Limitations and outlook", we point at the apparent mismatch between the firing statistics in our model and those found in nature, and explain how a more natural irregular activation of sequences, parallel processing, and subsampling would resolve this mismatch. We illustrate these arguments with a new Supplementary Figure S5. In addition, we discuss other future extensions of the model, such as working memory, recurrent dynamics in inhibitory populations, and background inputs, that would contribute asynchronous irregular activity in the TM model.

[R1:4] *Both these two features are highly relevant and, given the presence of plasticity and the feedback inhibitory loop, might significantly alter the model dynamics. To facilitate biological interpretation it would therefore be important to show that the network can learn and reproduce high-order stimulus sequences also after the introduction of these two features.*

As described in the new paragraph in the Discussion (see [R1:3]), irregular external inputs,

parallel processing and subsampling would mitigate the apparent mismatch between the natural firing statistics and those predicted by our model. Provided the conditions described in section "Results: Dependence of prediction performance on the sequence speed" are fulfilled, irregular inputs would barely affect the plasticity and WTA dynamics, and still lead to high learning and prediction performance. Subsampling is a mere measurement argument and does not affect the model dynamics. We think that learning multiple sequences in parallel is possible provided there is no systematic relationship between sequences across trials (otherwise, these sequences would be merged).

The scope of our work is a demonstration that the original TM model proposed by Hawkins & Ahmad (2016) can be implemented using biological ingredients without performance losses. To foster comparability, we designed the model and the performance evaluation protocols such that they are as close as possible to those used by Hawkins & Ahmad (2016). We think that an explicit demonstration of the ideas described above is beyond the scope of this work, and should rather be the subject of follow-up studies.

[R1:5] *There is a vast literature on: a) neural networks able to produce sequences, b) networks able to produce replays (backward and forward). Surprisingly the section on 'Relationship to other models' doesn't address this literature, as one would expect, except for the sentence "This is in essence similar to several other spiking neuronal network models for sequence learning [9–12].". It would be useful to add a paragraph where other models are discussed and compared with the proposed model.*

We agree. In the original version of our manuscript, we provided a list of other related studies, but discussed only some of them in more detail, for example, when describing the role of dendritic action potentials. In the revised manuscript, we have now expanded the text in "Discussion: Relationship to other models" to make the similarities and differences between our and other models more explicit. We specifically discuss the work of Maes et al. (2020) and Cone & Shouval (2021) providing solutions for the learning of sequence timing, the model of Asabuki & Fukai (2020) and Asabuki & Fukai (2021) used for context specific feature segmentation, and the work of Schulz et al. (2021) on the generation of novelty (mismatch) responses (see also our replies to points [R2:1], [R2:2] and [R2:3] of reviewer 2).

[R1:6] *Minor points:*
The model has one inhibitory neuron for each excitatory subnetwork. Fig 2A,C could lead to interpret the inhibitory network as recurrently coupled with the full network of excitatory neurons. I would suggest to slightly modify the scheme and add as many inhibitory neurons as the excitatory subnetworks, each of them connected only to their respective excitatory subgroup.

We agree that the original figure was misleading. We have now adapted this figure and indicate that the inhibitory population consists of stimulus specific interneurons. In addition, we emphasize in the caption that these interneurons are specifically connected to their respective excitatory subpopulations.

[R1:7] *There is a probable typo at line 236: "...synaptic depression, and is triggered by each presynaptic spike ...", Most probably the authors mean postsynaptic.*

No, this is not a typo. We use the event based STDP update scheme proposed by Morrison et al. (2007), where depression is indeed triggered at presynaptic spike times.

[R1:8] *Line 137: "connections can be either "mature" or "immature" ("effective" or "potential"; see below)". For clarity, I would stick with one of the two definitions throughout the manuscript (either mature/immature or effective/potential). Also, immature/potential connections, although updated, do not affect units activity when running the network. Even though this is deducible from lines 197- 198, it is not evident from the rest of the paper. I would suggest adding this information explicitly, at least at line 137, when the two types of connections are introduced.*

We agree that these terms were not well defined and not consistently used in the original manuscript. In the revised paragraph "Methods: Network model: Network structure", we now make clear that a potential connection can be either mature or immature, and explicitly mention that an immature connection has no effect on the target neuron. In the remainder of the manuscript, we refer to "potential" connections only in this sense, and otherwise use "mature" and "immature" consistently.

[R1:9] *When referring to "relative number of active neurons", e.g. legend of Fig. 8C, to my understanding the authors refer to the relative number of active neurons of the targeted population. If this is the case, it would be useful to add this information in the label of the plot axis or at least in the figure legend.*

We thank the reviewer for pointing this out. The reviewer is right that the reported numbers of active neurons are measured in units of the subpopulation size. We adapted the figure captions accordingly.

Reply to comments of reviewer #2

We thank the reviewer for the valuable comments and the constructive critique. In the revised version of the manuscript, we have addressed all the reviewer's concerns, and marked all revised parts by color and a reference "[R2:X]" to the corresponding comment X. A point-by-point reply is provided below.

[R2:1] *Major questions:*
While combining several tasks is a feature of the model, previous work focusing on individual tasks may have the benefit that they can zoom in and mechanistically understand things in great detail. It seems that some part of the solution or implementation in these other studies could be shared with the solution in this paper. In this regard, you already cited resemblance with amongst others Cone and Shouval, eLife 2021, the sequence learning aspect. A more detailed discussion may be beneficial for the reader.

We added more details to the section "Discussion: Relationship to other models", discussing other sequence learning models, in particular the works by Cone & Shouval (2021) and Maes et al. (2020), and how their ideas could be combined with ours. In contrast to our model, the model of Maes et al. (2020) learns not only the sequence order, but also sequence specific timing and duration of individual elements. To this end, they introduce and train an additional clock network. We propose that our model could be extended in future work by adding plastic recurrent connections within each excitatory subpopulation, thereby adding this functionality without the need for an additional auxiliary network. Moreover, we point out that the TM model represents a more efficient way of learning high-order sequences, and that the underlying mechanism could be included in the models of Cone & Shouval (2021) and Maes et al. (2020).

[R2:2] *As the authors are also interested in mismatch/prediction it would be good to discuss resemblance in this domain, for example: Schulz et al., eLife 2021.*

We extended the paragraph on the mismatch detection in "Discussion: Relationship to other models", discussing the work of Schulz et al. (2021) and indicating similarities and differences. We point to the inhibitory feedback as a mechanism required for both models to generate sparse activity in response to a familiar stimulus. We emphasize, however, that the scope of the two studies is different. As opposed to the model by Schulz et al. (2021), which learns only a set of elements, our model learns the order of sequence elements in a context dependent manner.

[R2:3] *Finally I am wondering if there is any link to feature segmentation, as studied by for example the group of Tomoki Fukai (example paper is Asabuki and Fukai, Nat Comms 2020).*

Sequence chunking as studied by Asabuki & Fukai (2020, 2021) refers to the ability of identifying recurring sequences within a long stream of inputs. It is possible that our model can also perform chunking if the recurring sequences are presented randomly and without breaks. This way the connections between neurons representing different sequences are not strengthened by spike-timing-dependent potentiation and therefore the different sequences remain separate. However, this processing ability needs to be tested in future studies. In a new paragraph in "Discussion: Relationship to other models", we address feature segmentation and chunking and further discuss how the work of Asabuki & Fukai (2020, 2021) relates to ours.

[R2:4] *Is there a limit to the history/context memory? For example would ABCDEFG and HBCDEFJ give different predictions for the final element? In other words, would the homeostasis*

still be able to separate the pathways?

We thank the reviewer for bringing up this example. In the TM model, each sequence element in a particular context activates a specific pattern. The number of different patterns that could be learned is determined by the size of each subpopulation and the sparsity. Without repeating elements within a sequence, the maximum order is only limited by the total number of subpopulations, i.e., the vocabulary size. We added a paragraph on the maximum sequence order at the end of the section: "Results: Prediction performance", and depict learning performance results for two sequences of order 10 in the Supplementary Materials (Fig. S3).

[R2:5] *Minor questions:*
Network structure: the M non-overlapping subpopulations are hardcoded into the exc neurons rnn? This merely means that they receive the same input, but the connectivity is still random?

The reviewer is right. To make this clearer, we improved the text in the paragraph "Methods: Network model: Network structure".

[R2:6] *External inputs: how long in time are individual elements? When for example sequence ABCD is given, does each letter trigger a single spike? What if it triggers multiple? I suppose the refractory period does not allow this.*

Yes, the reviewer is right. The occurrence of a sequence element triggers a single spike in each neuron participating in the corresponding subpopulation. Secondary spikes would mainly be avoided by the inhibitory feedback. In principle, multiples spikes per neuron are not a problem as long as the activity remains spatially sparse and the initial response is synchronous. See also our answers to comments [R1:2,3] of reviewer 1.

[R2:7] *Equation 1: x_j is written after the summation and y_i before. For esthetic reasons I would put both either before or after.*

We thank the reviewer for pointing this out. We followed the advise and revised the equation accordingly.

[R2:8] *Line 223: why are the minimal permanences uniformly distributed? Is this how they are initialized, and hence guarantee the necessary heterogeneity?*

Yes, distributing the minimal permanences is necessary to provide a form of heterogeneity that persists over time. Heterogeneity is a prerequisite for the emergence of context dependent neuronal activation (see "Results: Sequence learning and prediction"). Heterogeneity in the initial condition is not sufficient as this would be washed out during the learning process, thereby prohibiting relearning of new sequences. We have slightly adapted the text in the paragraph "Methods: Network model: Plasticity dynamics" to clarify this point.

[R2:9] *Are the parameters, for example dAP threshold of 59pA etc, taken from a particular previous study or finetuned?*

The majority of parameter values has been chosen to be compatible with biology in the sense that they are of the right order of magnitude (some exceptions are mentioned in the "Discussion: Limitations and outlook") or lead to phenomenological behavior that matches biology. The dAP

threshold, for example, has been chosen such that the co-activation of 5 presynaptic neurons with mature connections to the target neuron reliably triggers a dAP. Experimental studies report minimal required numbers of coincident inputs in the range 10–20 (Major et al., 2013; Mengual et al., 2020), which is only slightly larger than the number we used in this study. We added information to the paragraph "Methods: Network model: Neuron and synapse model" clarifying this point.

[R2:10] *What is the purpose of the recurrent EE connectivity? Am I correct in saying that the feedforward pathways, being carved out, is what makes the model work?*

We thank the reviewer for pointing this out. In the revised manuscript, we have now added a new paragraph in "Discussion: Relationship to other models" on the definition of "recurrence" and it's functional role in the TM model. Moreover, we removed the term "feedforward" mostly everywhere in the manuscript, because the sequence specific subnetworks carved out by the plasticity dynamics are not strictly "feedforward".

References

Hawkins, J., & Ahmad, S. (2016). Why neurons have thousands of synapses, a theory of sequence memory in neocortex. *Frontiers in neural circuits 10*, 23.

Maes, A., Barahona, M., & Clopath, C. (2020). Learning spatiotemporal signals using a recurrent spiking network that discretizes time. *PLoS computational biology 16*(1), e1007606.

Cone, I., & Shouval, H. Z. (2021). Learning precise spatiotemporal sequences via biophysically realistic learning rules in a modular, spiking network. *Elife 10*, e63751.

Asabuki, T., & Fukai, T. (2020). Somatodendritic consistency check for temporal feature segmentation. *Nature Communications 11*(1), 1–13.

Asabuki, T., & Fukai, T. (2021). Neural mechanisms of context-dependent segmentation tested on large-scale recording data. *bioRxiv*.

Schulz, A., Miehl, C., Berry, M. J., & Gjorgjieva, J. (2021). The generation of cortical novelty responses through inhibitory plasticity. *eLife 10*, e65309.

Morrison, A., Aertsen, A., & Diesmann, M. (2007). Spike-timing dependent plasticity in balanced random networks. *Neural Comput. 19*, 1437–1467.

Major, G., Larkum, M., & Schiller, J. (2013). Active properties of neocortical pyramidal neuron dendrites. *Annu. Rev. Neurosci. 36*, 1–24.

Mengual, U. M., Wybo, W. A., Spierenburg, L. J., Santello, M., Senn, W., & Nevian, T. (2020). Efficient low-pass dendro-somatic coupling in the apical dendrite of layer 5 pyramidal neurons in the anterior cingulate cortex. *Journal of neuroscience 40*(46), 8799–8815.
