# Supplementary material for: Sequence learning, prediction, and replay in networks of spiking neurons
Source: PLoS Comput Biol. 2022 Jun 21;18(6):e1010233. doi: 10.1371/journal.pcbi.1010233 (PMC9273101; doi:10.1371/journal.pcbi.1010233)
Supplement: Attachment — Submitted filename: reply_letter.pdf [file pcbi.1010233.s013.pdf]

Younes Bouhadjar
Institute of Neuroscience and
Medicine (INM-6) &
Peter Grünberg Institut (PGI-7)
Jülich Research Centre
D-52425 Jülich
Germany

phone: +491778728402
email: y.bouhadjar@fz-juelich.de

Stefan Kiebel and Samuel Gershman
Editorial Board Member, PLoS Computational Biology

Jülich
April 28, 2022

Resubmission of manuscript PCOMPBIOL-D-21-02013

Dear Stefan Kiebel and Samuel Gershman,

please find enclosed our revised manuscript *"Sequence learning, prediction, and replay in networks of spiking neurons"*, which we resubmit for publication in PLoS Computational Biology. The concerns of the reviewer have been addressed, and the manuscript was changed accordingly. For the revised version, we ran the simulations requested by the reviewer, thereby demonstrating robustness of the proposed model with respect to a moderate level of synaptic background noise as well as non-synchronous inputs, but also making the limitations more explicit.

Best regards,

Younes Bouhadjar (on behalf of all authors)

encl
- reply to reviewer
- revised manuscript with highlighted changes
- revised manuscript without change highlighting

Reply to comments of reviewer #1

[R1:1] *As outlined in the major points of my previous review, the activity produced by the model is extremely far from what is observed experimentally. Input stimuli are passed to the network by one single spike; there is no rate modulation, neither in the input signal nor in the response of the network; and the model does not display any background activity. These are extreme conditions, and leave the reader wondering how the model would function in a more realistic scenario. Therefore, I had suggested new simulations. For example, a first quick way to address some of these concerns could have been to re-run the existing model (learning and prediction) with input signals that include (beyond the sequences to learn) also noise spikes and/or rate modulation. Such analyses are quick to perform, as they do not require any modification in the model, and their outcome, either positive or negative, would be highly informative. The authors did not perform new analyses but addressed the concerns by adding a paragraph in the Discussion of the manuscript (and a new Suppl. Fig. 5) where they discussed the degree of synchronization present in experimental data and how synchrony might go unnoticed when subsampling. In their reply letter the Authors write: "In a new paragraph added to "Discussion: Limitations and outlook", we point at the apparent mismatch between the firing statistics in our model and those found in nature, and explain how a more natural irregular activation of sequences, parallel processing, and subsampling would resolve this mismatch". In my opinion, this does not resolve the mismatch and does not address the concern raised. Moreover, this line of argumentation (together with the addition of Suppl. Fig. 5) appears to suggest that brain activity 'is' exclusively composed of synfire chains. While there is large experimental evidence of the presence of highly synchronized neuronal ensemble in the brain, assuming that such kind of activity constitutes the totality of the neuronal activity is a very strong claim. Therefore, in my opinion it is necessary to perform additional simulations, on the line of those described above, to provide insightful addition to the field suited for PLOS Computational Biology.*

We welcome the reviewer's constructive critique, and ran the suggested simulations with a) dispersed, non-synchronous inputs, and b) synaptic background noise. The results are presented in the new Supplementary Figures S6 and S7. In addition, we added new text on the effects of non-synchronous inputs and background noise on the network performance to "Discussion: Limitations and outlook".

As suggested by the reviewer, we first tested whether the spiking TM model critically relies on the assumption that the external inputs are presented in the form of perfectly synchronous spikes. To this end, we replaced the external inputs by volleys of spikes with a certain spike-time dispersion (see caption of Figure S6 for a more detailed description). The results show that sequences represented by non-synchronous inputs are learned and processed with low error provided the spike responses of the target populations remain nearly synchronous (see explanation in "Discussion: Limitations and outlook"). In the absence of background noise, the spike-time dispersion in the inputs is hence not critical. For a larger input dispersion, one would need either more spikes or larger synaptic weights to guarantee firing of the target neurons. With additional background noise (see below), a dispersion in input spikes could easily lead to a high dispersion in response spikes. The results of the new simulations show, however, that the dispersion in the response spikes remains low for moderate levels of background noise and a small dispersion in input spikes, such that the network performance is preserved. The new text in "Discussion: Limitations and outlook" suggests potential future adaptations of the model enabling the learning and processing of sequences for external inputs with a larger dispersion.

We further tested to what extent the spiking TM model can cope with background noise mimicking synaptic input from large populations of excitatory and inhibitory neurons firing in an asynchronous, irregular manner with realistic rates (see caption of Figure S6 for a more detailed description of the noise model). The simulation results show that the model performance is robust with respect to moderate levels of background noise (Figure S7). High levels of input noise (larger weights of background inputs) leading to additional, non-task related "background" spikes, however, overwrite the dAP triggered depolarization, which is required for successful sequence learning and processing. We conclude that the spiking TM model is compatible with membrane potential flucuations observed in the in-vivo activity (such as those reported by DeWeese et al., 2006, J Neurosci 26). It cannot explain the generation of irregular, non-synchronous firing by random, high-amplitude background noise. However, as outlined in the "Discussion", there exist multiple potential alternative mechanisms or settings leading to irregular, asynchronous firing of neurons in the TM model (such as a working memory or parallel processing of multiple sequences with irregular inter-element intervals).
