# Supplementary material for: Sequence learning, prediction, and replay in networks of spiking neurons
Source: PLoS Comput Biol. 2022 Jun 21;18(6):e1010233. doi: 10.1371/journal.pcbi.1010233 (PMC9273101; doi:10.1371/journal.pcbi.1010233)
Supplement: Attachment — Submitted filename: reply_letter.pdf [file pcbi.1010233.s014.pdf]

Younes Bouhadjar
Institute of Neuroscience and
Medicine (INM-6) &
Peter Grünberg Institut (PGI-7)
Jülich Research Centre
D-52425 Jülich
Germany

phone: +491778728402
email: y.bouhadjar@fz-juelich.de

Stefan Kiebel and Samuel Gershman
Editorial Board Member, PLoS Computational Biology

Jülich
May 19, 2022

Resubmission of manuscript PCOMPBIOL-D-21-02013

Dear Stefan Kiebel and Samuel Gershman,

please find enclosed our revised manuscript *"Sequence learning, prediction, and replay in networks of spiking neurons"*, which we resubmit for publication in PLoS Computational Biology. The concern of the reviewer has been addressed, and the manuscript was changed accordingly.

Best regards,

Younes Bouhadjar (on behalf of all authors)

encl
- revised manuscript with highlighted changes
- revised manuscript without change highlighting
